# Advanced Computer Simulation Based on Cardiac Imaging in Planning of Structural Heart Disease Interventions

**DOI:** 10.3390/jcm14196885

**Published:** 2025-09-29

**Authors:** Alaukika Agarwal, Lauren Ranard, Torsten Vahl, Omar Khalique

**Affiliations:** 1 Department of Medicine, Staten Island University Hospital, New York, NY 10305, USA; 2 Division of Cardiology, Columbia University Medical Center, New York, NY 10032, USA; 3 Division of Cardiovascular Imaging, St. Francis Hospital and Catholic Health, Roslyn, NY 11576, USA

**Keywords:** computer simulation, structural heart interventions, transcatheter aortic valve implantation, finite element analysis, left atrial appendage occlusion, patient-specific modeling

## Abstract

The rapid expansion of structural heart interventions over the past decade has created unprecedented challenges in procedural planning and complication prediction. While traditional imaging provides essential anatomical information, translating two-dimensional images into a comprehensive three-dimensional understanding of complex cardiac structures remains challenging. This review encompasses finite element analysis (FEA), computational fluid dynamics (CFD), and fluid–structure interaction (FSI) technologies across major structural heart procedures, including transcatheter aortic valve implantation (TAVI), transcatheter mitral valve interventions, and left atrial appendage occlusion (LAAO). We evaluated the technical foundations, clinical validation studies, and practical applications of various simulation platforms. Advanced computer simulation has demonstrated feasibility and clinical utility across multiple structural heart procedures. Computer simulation for structural heart interventions has evolved from a proof of concept to clinical implementation, with growing evidence of procedural planning benefits in TAVI and LAAO. While feasibility has been established across multiple intervention types, this field requires larger validation studies to demonstrate accuracy and clinical outcome improvements. Future directions include integration of machine learning, real-time simulation capabilities, and expanding applications to complex anatomies and redo procedures. This technology represents an emerging paradigm that may facilitate precision medicine in structural heart interventions, with potential for significant improvements in procedural success and patient safety.

## 1. Introduction

There has been exponential growth in structural heart disease interventions over the past decade [1,2]. The landmark approval of transcatheter aortic valve implantation (TAVI) with the Edwards SAPIEN^TM^ transcatheter heart valve in 2011 led to the rapid progression of this field, with subsequent expansion addressing different pathologies across all cardiac valves and structural components [3,4]. This further led to a proliferation of novel devices, new innovative techniques, and expanding indications, creating a need for clinicians to optimize patient outcomes [5].

There are several factors that compound the complexity of structural heart interventions, including significant anatomical variability among patients and procedural decision making [6]. This differs from traditional open surgical approaches, where visualization and tactile feedback guide interventions. Instead, transcatheter approaches rely heavily on advanced imaging for procedural planning and execution [7]. Subsequently, there has been an evolution in the role of imaging in structural heart interventions beyond initial diagnosis to include screening for treatment selection, device sizing, and prediction of potential complications [8,9].

Pre-procedural planning has progressed to encompass multimodality imaging as the cornerstone of patient assessment [10]. Computed tomography (CT), echocardiography (echo), and cardiac magnetic resonance imaging (CMR) supply data regarding cardiac structure, function, and hemodynamics that inform procedural strategy. However, translating these images into a comprehensive three-dimensional understanding of complex cardiac structures remains challenging [11,12].

To address this challenge, three-dimensional printing has emerged as one approach to create physical representations of patient-specific cardiac anatomy [13]. These models enable hands-on simulation and device testing before the actual procedure, potentially reducing the procedural time and complications [14]. Despite these advantages, 3D printing has significant limitations. The process is costly, time-consuming, and labor-intensive, which restricts its routine clinical application [15]. More importantly, physical models typically represent static anatomical states and fail to capture the dynamic motion of cardiac structures throughout the cardiac cycle [16,17]. Additionally, the material properties of 3D-printed models cannot accurately replicate the biomechanical characteristics of cardiac tissues, which limits their utility in predicting tissue–device interactions [18,19].

Prior reviews of the simulation space have focused on specific procedures [20] or have broadly discussed many subjects, such as AI and 3D printing [17,21,22]. This review adds to recent publications by focusing in depth on the specific topic of advanced computational modeling across the breadth of structural heart interventions. This review discusses computational techniques such as finite element analysis (FEA), computational fluid dynamics (CFD), and fluid–structure interaction (FSI) methodologies, which are specifically applied to imaging-derived cardiac models. The primary focus is a review of imaging-derived advanced computer simulation techniques and the literature within the structural heart disease space.

## 2. Rationale for Advanced Computer Simulation

These limitations highlight the importance of computational modeling in structural heart interventions. Computer simulation offers several advantages over physical modeling, enabling the creation of patient-specific dynamic models that represent cardiac structures throughout different phases of the cardiac cycle [23]. Virtual devices can be deployed within these models to predict interactions with patient anatomy while accounting for tissue characteristics, flow parameters, regional calcifications, and structural deformation [17]. While basic imaging predictors for many of the typical procedural complications are well known within the literature, their positive and negative predictive values are modest, which suggests interpatient variability and the need for patient-specific modeling. These simulations can theoretically anticipate potential complications, optimize device selection and positioning, and ultimately improve procedural outcomes [20].

The integration of these computer simulation tools into clinical practice has been facilitated by the development of specialized software platforms tailored to specific structural heart interventions [21]. These platforms vary in their capabilities, accessibility, and validation, creating opportunities and challenges for clinicians seeking to implement simulation-based planning in their practices [24].

**Figure 1:**
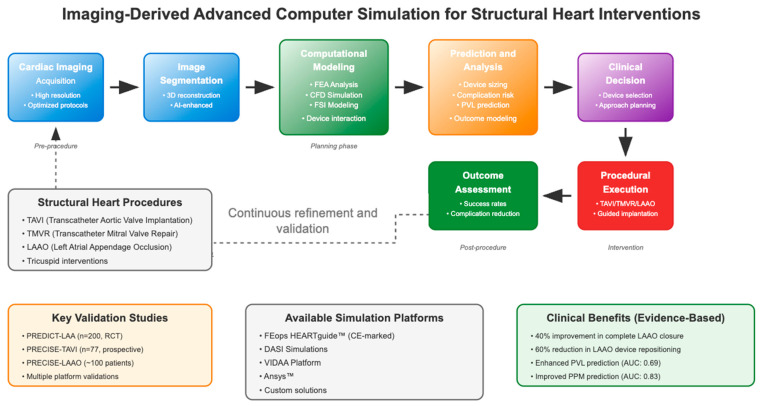
Integrative clinical workflow for imaging-derived simulation in structural heart interventions, from initial imaging and computational modeling to clinical outcomes and continuous refinement.

This paper provides a comprehensive overview of current computer simulation technologies for structural heart interventions, examining their technical foundations, clinical applications, and future directions. Through this analysis, we aim to elucidate the potential for computational modeling to advance the field of structural heart interventions while acknowledging the practical constraints that may influence its adoption in routine clinical practice (Figure 1).

### Clinical Translation and Validation Evidence

Clinical translation of cardiac simulation has demonstrated a measurable impact through several landmark validation studies. The PREDICT-LAA randomized controlled trial (*n* = 200) represents the first prospective, randomized evidence that AI-enabled computational modeling significantly improves procedural outcomes, showing 40% more complete LAA occlusion (61.1% vs. 44.0%, *p* = 0.03), a 60% reduction in repositioning devices > three times (10.0% vs. 22.7%, *p* = 0.02), and 80% fewer device retraction requirements (3.0% vs. 16.5%, *p* < 0.01) compared to standard planning [25]. Similarly, the PRECISE-TAVI study validated FEA predictions of paravalvular leak (PVL cutoff of 12.2 mL/s, effectively discriminating more than or equal to trace PVL and less than trace PVL with an AUC of 0.69) and conduction disturbances (contact pressure index > 11.5, predicting a permanent pacemaker with an AUC of 0.83) in challenging anatomies [26]. Commercially available platforms are increasingly being developed with the goal of minimizing TAVR-related complications and associated healthcare costs.

## 3. Types of Computer Simulations and Platforms

### 3.1. Basic Computer Modeling

**Figure 2:**
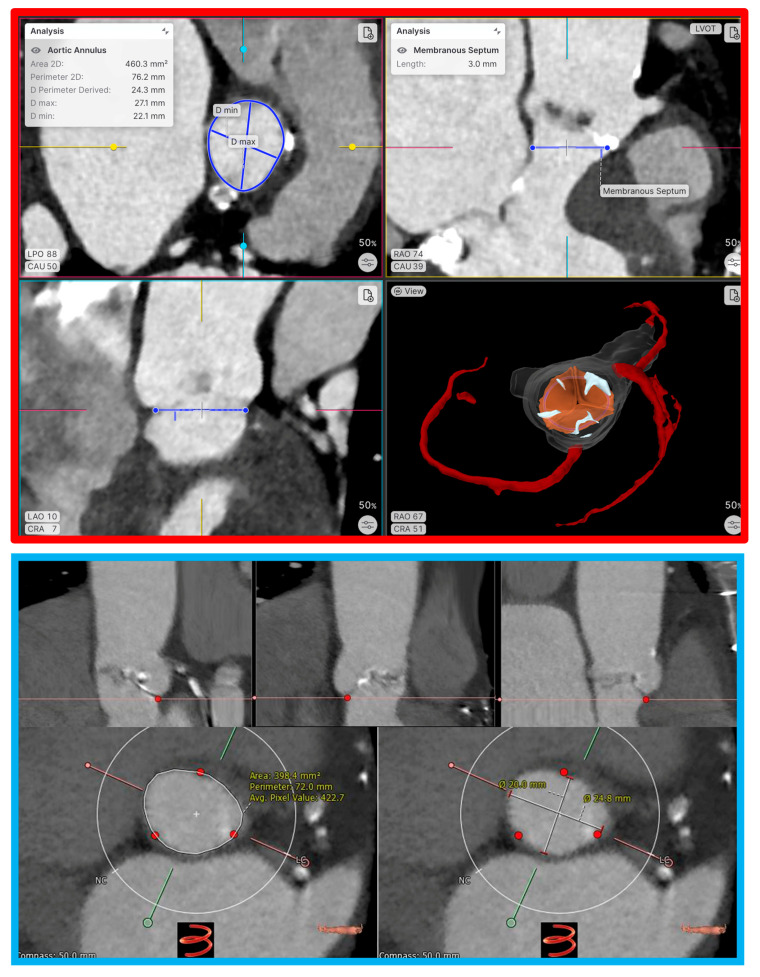
Basic segmentation and modeling of the aortic root. **Red panel**: The aortic root was auto-segmented, and basic measurements of the aortic annulus dimensions were derived using Laralab’s heart.ai software (Munich, Germany, https://www.laralab.com/, accessed on 15 July 2025). **Blue panel**: Automated segmentation was performed by detection of each aortic valve leaflet nadir, and aortic annulus measurements were performed using 3mensio^TM^ (Pie Medical Imaging, Maastricht, The Netherlands).

Basic computer modeling focuses primarily on anatomical visualization and measurement, providing fundamental insights for device sizing and procedural planning [27]. Basic computer modeling has existed since the early days of TAVI and consists of an embedded geometrical shape inserted into a cardiac computed tomography angiography (CCTA) image. This type of modeling can be used to determine the basic fit of a transcatheter valve in the target zone. A simple example of this is modeling a cylinder of the predicted THV size for TAVI. Examples of vendors in this space include 3mensio^TM^, Circle Cardiovascular Imaging, and Laralab (Figure 2).

### 3.2. Advanced Computer Simulation

Advanced simulation typically involves the creation of a digital twin of the structure(s) in question and use of finite element analysis (FEA) to predict tissue deformation and responses to interventions. There is research on individual centers or labs predicting implant success. In the past several years, vendors dedicated to advanced simulation have emerged. Examples of technologies in this space include FeOPS Heart Guide^TM^ and DASI simulations. Some technologies also model flow, which typically involves performing computational fluid dynamics (CFD) and fluid–structure interaction (FSI) analyses in a gap region or area from the FEA. Examples of available platforms include FEOPS^TM^, DASI, VIDAA, and Ansys^TM^. This review will focus primarily on advanced computer simulation.

### 3.3. Platform–Device Compatibility

Current simulation platforms demonstrate varying capabilities across device families and procedures, spanning TAVI, LAAO, and emerging TMVR applications. This is largely dependent on collaborations with device manufacturers, who can provide key material properties and device designs in order to facilitate computer simulations. Without adequate info, computational simulation platforms become less accurate. Additionally, given the workload for computational heavy lifting, the platforms tend to focus on a limited number of procedures and thus self-limit their portfolios of devices (Table 1).

## 4. Computer Simulation for Specific Structural Heart Interventions

**Figure 3:**
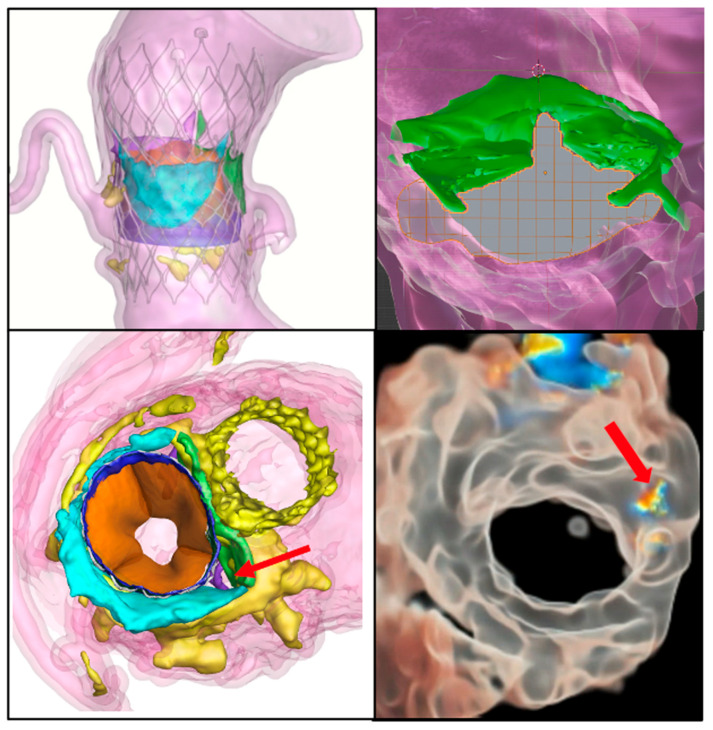
Computer simulation of TAVI and mitral valve interventions. **Upper left**: Simulation of tissue interaction for an S3 in an Evolut redo TAVI. **Upper right**: A 3D mesh with simulation of anterior mitral leaflet laceration using electrosurgery. **Lower left**: Simulation of a valve in an MAC procedure, predicting residual paravalvular leak in the mitral commissure (red arrow). **Lower right**: An actual post-procedural 3D color Doppler TEE image demonstrating paravalvular leak in the same commissure that was predicted. The images were provided by DASI simulations, Dublin, Ohio.

Compared to traditional surgical repairs of valves, transcatheter valvular interventions provide minimally invasive alternatives to patients with valvular heart disease. Transcatheter procedures encompass unique risks compared to open heart surgery. Computer simulation models attempt to mitigate these risks by ensuring procedural planning that is specific to patients, assisting in optimal device selection, and predicting complications (Figure 3).

### 4.1. TAVI

As TAVI was the first approved transcatheter valve procedure, the majority of the advanced computer simulation literature centers around it.

#### 4.1.1. Optimal Sizing

The major TAVI vendors have well-developed sizing charts to address different ranges. Basic computer simulation can be applied to cases to visualize a static valve within the segmented aortic root. In current practice, this is the workhorse method for most TAVI programs.

#### 4.1.2. Anatomical Rupture

Aortic root rupture (ARR) is a rare but serious complication of TAVI with a mortality rate that has been reported to be as high as 48% [29]. This is in a continuum with paravalvular leak (PVL), as overexpansion of a balloon or valve in the presence of severe calcification may lead to root rupture, whereas underexpansion can lead to PVL. The difficulty in predicting ARR is evidenced by the dearth of research on the subject. In 2013, Barbanti et al. stated that ≥20% prosthesis oversizing of a balloon-expandable valve and moderate/severe LVOT calcifications were predictors of ARR [29]. In 2017, Girdauskas et al. described six patients who had ruptured their LVOT in the anatomically weaker area between the left fibrous trigone and the left–right commissure within the muscular LVOT [30]. In theory, computer simulation would benefit prediction of the rare but morbid complication of ARR. There is only one computer simulation study in the literature. It was performed by Wang et al., who employed finite element analysis on one retrospective ARR case and two prospective cases [31]. The results were mixed, again highlighting the real-world difficulties in understanding this complex phenomenon.

#### 4.1.3. Paravalvular Leak

Aortic post-TAVI PVL has become less common over time, particularly PVL of moderate or greater severity. However, mild PVL is common, and some analyses have demonstrated increased morbidity and/or mortality, even at mild levels [32]. Basic predictors are well known, including transcatheter heart valve (THV) undersizing, the THV type (it is more common in self-expanding valves), significant calcifications, valve positioning, and a native bicuspid valve. However, there is significant interpatient variability. Many retrospective, observational studies have shown the ability to predict PVL retrospectively from computer simulations [33,34,35,36,37]. Mao et al., Bianchi et al., and Ghosh et al. demonstrated the feasibility of using FEA or FEA-FSI modeling to predict PVL based on the orientations and deployment depths of THVs [38]. Luraghi et al. and Nappi et al. demonstrated the effects of calcium on PVL formation [35,37]. Hokken et al. demonstrated an AUC of 0.69 for prediction of PVL after Evolut TAVI, with a simulated flow rate cutoff of 12.2 mL/s to predict > trace PVL [26].

#### 4.1.4. Percutaneous Alternatives for Coronary Artery Obstruction

Coronary obstruction (CO) is yet another rare but morbid complication of TAVI. Known anatomical risk factors for native TAVI based on basic modeling studies include a low coronary height of <12 mm and a low sinus of Valsalva diameter of <30 mm [39], and a low valve-to-coronary (VTC) distance of <4 mm [40]. For valve-in-valve (VIV) TAVI, known anatomical risk factors include a VTC of <4 mm and externally sewn bioprosthetic surgical valve leaflets. There has been very little work using advanced computer simulation to predict the risk of coronary obstruction. Heitkemper et al. studied a retrospective cohort of TAVI patients and used FEA to demonstrate that the VTC distance indexed to the coronary diameter was more predictive of CO than the typical metrics of coronary height and sinus of Valsalva diameter [41]. Holst et al. recently reported the feasibility of prospective use of CO FEA computer modeling prior to TAVI cases. In 29 TAVI cases with high predicted CO risk, 21 underwent a procedure or protective maneuver to avoid coronary obstruction, and none of the patients experienced CO [42].

#### 4.1.5. Leaflet Thrombosis

Leaflet thrombosis is a common sequela of TAVI, with a reported prevalence as high as 30–40%. Leaflet thrombosis appears to be more common in THVs than in surgical bioprosthetic valves. Known anatomical risk factors include a native bicuspid valve, moderate or severe PVL, a large anatomy, and the THV type. Attempts to predict leaflet thrombosis to date have focused on CFD analysis of stagnation of blood and/or neosinus washout [38,43,44,45]. Reduced neosinus washout and blood stagnation have been associated with thrombus formation in computational studies. Extensive clinical validations have not been performed.

#### 4.1.6. Prediction of Conduction Disturbances

In an initial FEA study by Rocatello et al., the metric of the contact pressure index (CPI), defined as the percentage of the area where contact between the prosthesis and the aorta generate a pressure exceeding 0.1 Mpa, predicted conduction disturbance at a threshold of 14%. However, only 26 patients in the 112-patient cohort were determined to have accurate predictions [46]. In a subsequent cohort of 56 patients with bicuspid or tricuspid aortic valve stenosis that were studied by the same group, the CPI was re-calibrated, which resulted in improved accuracy [47]. In a univariate analysis, only the CPI predicted a new-onset conduction disturbance. The traditional metrics of the oversizing ratio, implantation depth, and difference between the membranous septum length and the implantation depth were not effective for this prediction. The CPI exhibited a higher AUC (0.804) when predicting a new-onset conduction disturbance than the traditional factors. The PRECISE-TAVI trial, a prospective, observational study of computational simulation of complex aortic valve anatomies, similarly demonstrated an AUC of 0.83 for prediction of permanent pacemaker implantation after Evolut-PRO implantation, with a CPI cutoff of 11.5 [26].

#### 4.1.7. TAVI in Bicuspid Aortic Valves

Bicuspid valve TAVI is a complex area. Current THVs are not designed specifically to treat bicuspid aortic valves. Retrospective validation of FEA and CFD simulations has been performed by several groups in small studies [48]. The PRECISE-TAVI trial prospectively studied a group of patients, 17 (22.1%) of which had a bicuspid valve morphology [26]. As mentioned previously, this trial demonstrated the predictive abilities of FEA for permanent pacemaker implantation and PVL. FEA prompted a change in procedural strategy in 35% of the patients in this study. Due to small numbers, the bicuspid aortic valve group was not studied separately.

### 4.2. TMVR

#### 4.2.1. Mitral Valve-in-Valve TAVI

Given the excellent outcomes for mitral VIV TAVI with a balloon-expandable THV designed for TAVI, basic simulation appears to be adequate for the majority of cases. The sealing zone of a failed surgical bioprosthesis provides an excellent landing zone, and the morbid complication of left ventricular outflow tract obstruction (LVOTO) is rare in comparison to other forms of TMVR.

#### 4.2.2. Mitral Valve in Ring and Valve in Mitral Annular Calcification

A mitral valve in a failed annuloplasty ring (ViR) and a valve in a native mitral annular calcification (ViMAC) are more complex forms of TMVR associated with less favorable procedural outcomes, such as LVOTO and higher mortality rates. These are currently treated using a balloon-expandable THV designed for TAVI. This suggests the need for advanced computer simulation as opposed to valve-in-valve TAVI. Although the feasibility of computer simulation was shown in early examples [49,50,51], progress in this field is limited and data are sparse.

#### 4.2.3. Native TMVR

Dedicated (designed for the mitral valve) TMVR devices for native mitral valve disease (mitral regurgitation without MAC) are mostly in the investigational space, with early CE Mark and FDA approvals. There are no available data on advanced computer simulation in this category.

#### 4.2.4. Mitral Edge-to-Edge Repair

The feasibility of FEA and FSI analysis of the mitral valve leaflet structure and mechanics has been shown by several groups [52,53]. Dabiri et al. developed a framework not only to simulate mitral valve geometry and flow dynamics but also to predict the results of mitral transcatheter edge-to-edge repair (m-TEER) [53]. This group used actual 3D echocardiographic datasets and computational data from the literature to model mitral geometries and simulate the results of m-TEER procedures, including MR and leaflet stress, with single and multiple m-TEER devices. Dabiri et al. demonstrated via FEA that different locations of m-TEER reduced MR and the mitral valve area by different degrees. They emphasized that machine learning (ML) has fewer limitations than FEA overall but requires much larger datasets for analysis. In a separate publication, Dabiri et al. demonstrated that an ML model for m-TEER simulation reduced the computational time from 6 h to less than 1 s [54].

Although there are limited real-world validation data, several proof-of-concept computer simulation studies have been performed. Prescott et al. performed an FEA simulation to confirm that m-TEER could treat MR and prevent posterior leaflet stress and chordal rupture in a hypertrophic cardiomyopathy patient [55]. Hart et al. retrospectively studied the accuracy of pre- and post-m-TEER 3D TEE datasets from two real patients compared to FEA simulations. The FEA simulations only differed by 0.7–0.9 mm in the locations of contours compared to the actual pre-m-TEER datasets [56]. In both patients, FEA simulation accurately predicted the severity and location of post-m-TEER MR. Messika-Zeitoun et al. published the largest simulation analysis for m-TEER computational FEA modeling to date [57]. These authors studied five patients who underwent m-TEER. There was good agreement between the actual post-procedural MR grade and the simulated regurgitant orifice area. However, the agreement between the post-procedural mitral valve area modeling and the mitral valve area measured using 3D TEE was poor, with only one of five patient simulations being accurate.

#### 4.2.5. Limitations in Transcatheter Edge-to-Edge Repair Simulation

Current TEER simulations are in their infancy. For the most part, straightforward and simple anatomies are the current focus. Even with the focus on simple anatomies, predictive accuracy has been mixed, as described above. Non-central and commissural TEER were found to be very prevalent, representing between 38 and 43% of the published data [58,59].

Finite element models experience bending difficulties near the commissures and cannot adequately simulate the complex dynamic interactions between commissural chordae and leaflet tissue during the cardiac cycle [60]. These limitations result in poor prediction accuracy for device trajectory correction and an inability to reliably predict single-leaflet device attachment risk during commissural repairs [60].

### 4.3. Left Atrial Appendage Occlusion

Data from the NCDR LAAO (left atrial appendage closure) registry has documented excellent procedural outcomes with commercially available LAAO devices in the United States, with an implantation success rate of >98%. Yet one in four patients has evidence of a peri-device leak after 45 days. Recent evidence suggests that even small PDLs (<5 mm) are associated with a higher rate of thromboembolic events; this has therefore further fueled interest in improving procedural planning to achieve complete LAAO [61].

LAA anatomy is complex, and individual anatomy is variable. CT-based computational simulation algorithms allow clinicians to depict device implantation and assess tissue–device interaction as a means to improve procedural efficacy and predict the feasibility of LAAO, peri-device gaps, and the correct device size. Computational fluid simulation models have also been used to predict device-related thrombosis (DRT).

#### 4.3.1. Methods of Analysis for CT-Based Computational Modeling of LAAO

FEops HEARTguide^TM^ (FEops Ghent, Belgium).

**Figure 4:**
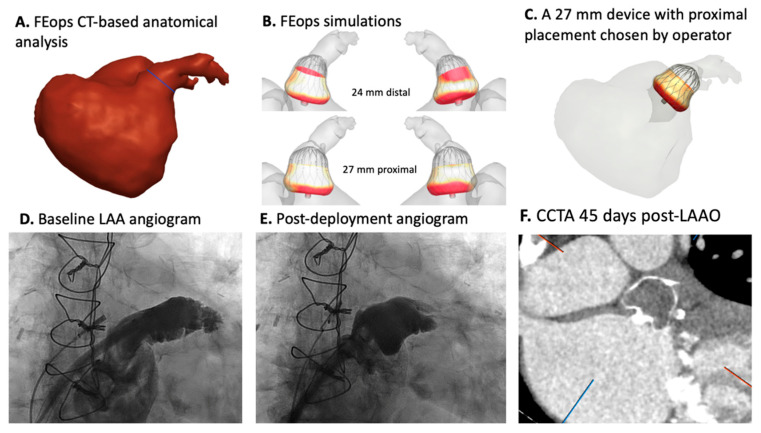
An example of the FEops^TM^ patient-specific computational model.

FEops HEARTguide^TM^ is a CE-marked and commercially available CT-based simulation technology that gives physicians insight into tissue–device interaction in patient-specific anatomy. Details of how the computational model was developed were previously published [62]. Briefly, computer-generated LAAO devices are virtually implanted into patient-specific anatomy using FEA computational simulation (Abaqus/Explicit finite element solver v170.0, Dassault Systems, Paris, France). Multiple sizes are simulated in different positions (proximal and distal), and frame deformation and device apposition are modeled. In addition, device diameters are assessed to evaluate compression. It is up to the provider to assess the simulations and select the most appropriate device size and position (Figure 4). The FEops^TM^ simulation technology has been validated for LAAO [50,63].

Multiple small to medium-sized prospective and retrospective studies have analyzed the utilization of FEops^TM^ for LAAO procedural planning (Table 2). The largest of these studies was the PREDICT—LAA study, which looked specifically at the value of FEops^TM^ for planning LAAO with the Amplatzer Amulet^TM^ device [25]. This was the first and, to date, only prospective, randomized trial of advanced computer simulation for device implantation. Though this study did not meet its primary endpoint (a composite of incomplete LAA closure with a residual grade III to IV distal leak and/or presence of device-related thrombosis on a post-procedural CT scan), there was a 31% decrease in the primary outcome in the FEops^TM^ group (28.9% in the FEops^TM^ group versus 41.8% in the standard treatment group, *p* = 0.08) and thus a trend in favor of CT-based simulation planning. Notably, the trial reached statistical significance (*p* < 0.05) for the secondary endpoints that focused on procedural efficacy (achievement of complete LAA closure, the number of devices used, the number of device repositionings, the procedural time, the radiation time, and the dye volume). Thus, overall, the trial demonstrated that the FEops^TM^ computational model offers improvements in procedural safety and efficacy for LAAO with the Amulet^TM^ device [25]. A few of the criticisms of this trial have been the lack of standardization in the control arm and the need for reproduction of the findings with a larger sample. Additionally, these findings cannot be extrapolated to other LAAO devices.

#### 4.3.2. Virtual Implantation and Device Selection in Left Atrial Appendages (VIDAA) Platform (Universitat Pompeu Fabra, Barcelona, Spain)

The VIDAA platform is a web-based 3D interactive virtual implantation application that allows a clinician to evaluate the LAA and manipulate LAAO device configurations to determine the optimal device selection. An individual’s anatomy is segmented based on a CT scan using 3D slicer and is used to create a 3D surface mesh. Both the CT scan and the surface mesh are then imported into the VIDAA program. CFD simulations are also performed. Two different LAAO devices are available in the platform: Watchman^TM^ and Amulet^TM^. The program proposes a set of appropriate LAA devices for a given geometry for the clinician to review. A catheter model is also available in the platform to simulate device delivery [67].

#### 4.3.3. Computational Fluid Dynamics to Predict Device-Related Thrombosis

CFD was first evaluated as a way to stratify the risk of LAA thrombus formation based on LAA morphology in atrial fibrillation patients. A number of studies have demonstrated that geometric characteristics of the LAA play a central role in defining thromboembolic risk [68,69,70,71]. Bosi et al. specifically looked at four different LAA morphologies (chicken wing, cactus, windsock, and cauliflower). First, CT scans were processed using Mimics (Materialise NV, Ghent, Belgium) to obtain the 3D anatomical shape of the LAA and LA. All 3D models were meshed and analyzed using Ansys^TM^ (Ansys^TM^, Inc., Canonsburg, Pennsylvania). CFD was evaluated in normal physiologic flow and pathological atrial fibrillation conditions. Blood flow patterns were characterized by their velocities and shear strain rates (SSRs). The velocity and SSR decreased from the ostium to the tip of the LAA for all LAA morphologies. In atrial fibrillation, the lowest velocity was observed with the cauliflower morphology and the highest was observed with the windsock morphology. Furthermore, in atrial fibrillation, the cauliflower morphology also showed the lowest SSR [69]. Thus, this study indicated that in pathologic conditions the cauliflower morphology is more prone to thrombus development. The cauliflower morphology has high thrombotic potential, as do morphologies that have multiple small lobes, which was confirmed in other studies [71,72].

Subsequently, CFD has been studied as a way to predict the risk of device-related thrombosis following LAAO. The most common measure evaluated is the endothelial cell activation potential (ECAP) index, which is an in silico thrombosis risk index. The ECAP combines the time-averaged wall shear stress with the oscillatory shear index. In general, low velocities (low wall shear stress values) and complex blood flow patterns (higher oscillatory shear index values) produce higher ECAP indices, which are thought to confer a higher risk of thrombus formation.

Table 3 details studies that have analyzed CFD for predicting DRT. Overall velocities < 0.2 m/s and an ECAP index > 0.5 Pa^−1^ have been suggested to be predictors of DRT. Another risk factor for DRT that emerged out of these studies is a low velocity along the pulmonary ridge if uncovered, especially if the pulmonary ridge flow cannot be washed out [73].

In the future, CFD has the potential to help individualize anti-thrombotic regimens post-LAAO. However, there are notable limitations to the CFD methodology that need to be addressed. There is no clear consensus on the optimal boundary conditions to model LA hemodynamics, and this needs to be standardized. This is especially important, as CFD has a high sensitivity to numerical assumptions. There is also a lack of model verification [78]. Lastly, this methodology has been evaluated in small studies and needs to be applied on a larger scale to further our understanding.

#### 4.3.4. Artificial Intelligence (AI) Integration and Enhanced Simulation

Integration of artificial intelligence with cardiac simulation accelerated significantly in 2023–2024. AI applications now span the entire cardiac simulation workflow from automated cardiac structure segmentation to predictive outcome modeling.

AI-powered clinical decision support systems integrate knowledge from basic biology to current pathophysiologic insights and drive real-time image analysis, segmentation, and annotation to guide operators in selecting the most appropriate strategies for individual patients [79]. Technical advances in cardiac simulation AI include physics-informed neural networks (PINNs) that incorporate cardiac biomechanics constraints, reducing CFD computation times while maintaining accuracy within traditional methods. AI-enhanced segmentation using U-Net architectures and automated mesh generation eliminate traditional post-processing artifacts while reducing processing times from hours to minutes [80].

However, significant challenges persist in AI implementation. Data quality limitations affect generalizability, with only 3.6% of FDA-approved AI devices reporting race/ethnicity data and 99.1% lacking socioeconomic information [81]. Deep learning models often operate as “black boxes” with limited interpretability, hindering clinical acceptance [82]. Technical challenges include the need for large, diverse training datasets and patient-specific anatomical variations that challenge model generalization [83]. Current solutions include Gradient-weighted Class Activation Mapping (Grad-CAM) for visualization and Shapley Additive Explanations (SHAP) for feature importance analysis, but standardized frameworks for AI model development and validation are lacking [84].

Current solutions and validation approaches include development of explainable AI frameworks specifically for cardiac simulation, using attention mapping to highlight anatomical features driving device sizing recommendations. Federated learning approaches allow model training across multiple institutions while maintaining patient privacy. However, standardized benchmarks for cardiac simulation AI performance are lacking, with validation studies typically involving single-center cohorts with relatively small samples of less than 200 patients.

#### 4.3.5. Implementation Barriers and Clinical Adoption Challenges

Despite technical advances, significant barriers limit widespread clinical adoption of cardiac simulation technologies. Cost barriers include high infrastructure requirements, including the costs of simulation centers, per-case costs, and substantial investments in specialized personnel and maintenance [85]. Additional expenses include specialized workstation hardware, high-resolution display systems, and training for dedicated technical personnel. Learning curve challenges persist. A survey conducted in 2024 found that 71% of interventional cardiology professionals reported inadequate training related to simulation-based planning, with most training experiences lasting under one week, which is insufficient for skill mastery [86].

Standardization issues represent a critical gap in cardiac simulation implementation. Currently, no standardized protocols exist for CT acquisition parameters optimized for simulation; mesh generation quality control; or simulation result interpretation involving data variability, protocol inconsistency, a lack of external validation, fragmented regulatory requirements, and limited interoperability among software systems [87,88,89].

Technical limitations include computational demands (CFD modeling requires many hours per case, increases memory requirements, and creates processing limitations, preventing real-time intraoperative guidance), moderate overall fidelity compared to actual clinical scenarios, and device-specific compatibility constraints [90,91].

Device and anatomy generalizability remain constrained. These limitations arise from platform-specific capabilities, with most simulation tools optimized for specific anatomical presentations and device families rather than comprehensive coverage of anatomical variations and pathological presentations [89,92], such as bicuspid valves, heavily calcified annuli, and prior surgical interventions, which often fall outside validated simulation parameters. This limits clinical applicability in complex cases, where simulation guidance would be most valuable.

## 5. Future Directions

Overall, this field needs more robust trials to prove the accuracy of advanced computational modeling for procedural simulation and, most importantly, translation to improvements in procedural and clinical outcomes. More computational advancement and power are needed to make simulations more efficient and allow timely intervention.

### 5.1. TAVI

**Figure 5:**
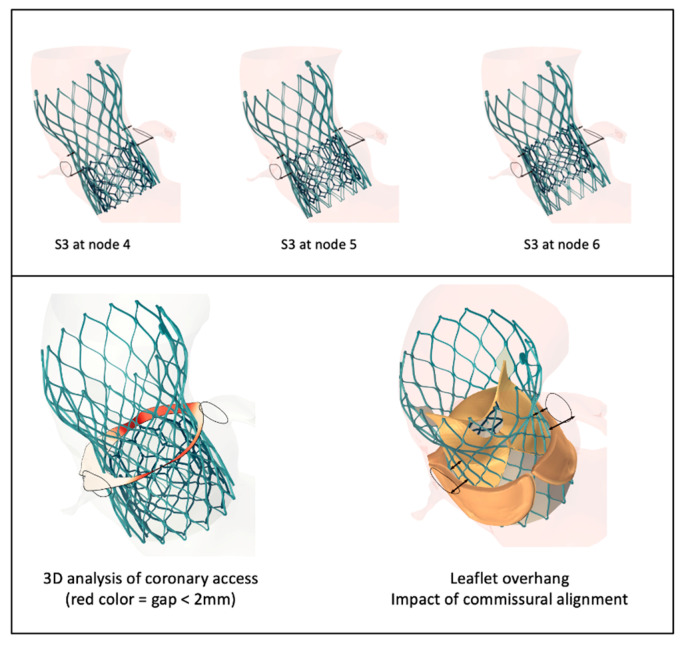
The feasibility of redo TAVI computer simulation. **Top panel**: Simulation of an S3 in an Evolut valve combination for redo TAVI at different implantation heights. **Bottom panel**: Simulation of a redo TAVI S3 in an Evolut combination, demonstrating the proximity to the aorta wall, leaflet overhang, and commissural alignment. The images were provided by FEops HeartGuide^TM^, Gent, Belgium.

As procedures become more complex, the need for advanced computational simulation will increase. The feasibility of redo TAVI (TAV in TAV) procedural simulation has been shown. However, more validation is needed (Figure 5). Lifetime management of aortic stenosis treatment (predicting results of sequential procedures) is another important area of need.

### 5.2. TMVR

Sizing is a concern in some valve-in-ring cases, particularly valve-in-MAC cases, and requires more accurate modeling. The displacement and behavior of MAC can be unpredictable with percutaneous valve implantation. However, the largest concern and most common exclusion for TMVR cases is LVOT obstruction. There is a great need for accurate computational modeling of LVOT obstruction, including the neo-LVOT area, tissue interactions, and displacement of the anterior mitral leaflet.

### 5.3. Tricuspid Interventions

Efforts are preliminary, but feasibility studies of computer simulation of tricuspid leaflet geometry have been performed. More work will be needed to validate procedural computational simulation as the field matures.

### 5.4. LAAO

While this is the only intervention that has a randomized, controlled trial as evidence, the results were only valid for a single device type. Computer simulation of other device types needs more validation.

## 6. Conclusions

The field of computational simulation for structural heart device implantation procedures has made significant progress. Feasibility has been proven. Future impacts in this field will be predicated upon validation of accuracy and improvements in outcomes.

## Figures and Tables

**Table 1:** Key clinical validation evidence for cardiac simulation platforms.

Procedure Type	Platform	Study Design	Sample Size	Key Outcomes	Clinical Impact
LAAO	FEops HEARTguide™	RCT (PREDICT-LAA) [25]	200 patients	Primary composite outcomes: 28.9% vs. 41.8% (*p* = 0.08, trend favoring simulation)	First AI-enabled RCT proving procedural improvement
LAAO	FEops HEARTguide™	Multicenter (PRECISE LAAO) [28]	~100 patients	Complete LAA closure: 59.8% with standard planning vs. 90.2% with simulation planning	Demonstrates improved procedural planning accuracy
TAVI	FEops HEARTguide™	Prospective (PRECISE-TAVI) [26]	77 patients	PVL prediction accuracy AUC: 0.69, PPM prediction AUC: 0.83	Validated predictive modeling for challenging anatomies

**Table 2:** Key findings of studies that have evaluated CT-based computational modeling for LAAO.

Publication	Study Design	Methodology	Key Findings
Buysschaert et al. [64] (2021)	Prospective study N = 15 Patients receiving LAAO with Amulet^TM^	Proceduralists first made sizing assessments based on standard pre-procedural planning and rated their confidence on a scale from 0 to 10. Then, they were provided the FEops^TM^ assessment and asked to make a sizing decision and re-rate their confidence in the decision.	Increase in confidence from 6.4 +/− 1.4 to 8.1 +/− 0.7 (*p* = 0.003) The final implanted size correlated with FEops^TM^ in 11/15 (73.3%) vs. 7/15 (46.7%) for standard care alone. The initial size decisions were changed after FEops^TM^ simulation in four cases. FEops^TM^ simulation correctly identified a lack of wall apposition in one procedure; nevertheless, this procedure was attempted but aborted.
PREDICT-LAA [25,65] (2023)	Prospective, multicenter, randomized study N = 200 Patients receiving LAAO with Amulet^TM^	Randomization (1:1) to computational simulation arm and standard arm Primary endpoint: Incomplete LAAC and DRT assessed at 3 months using post-procedure CT	Primary endpoint: 41.8% in the standard arm vs. 28.9% in the FEops^TM^ arm (*p* = 0.08). Secondary endpoints: Leak grades 3 or 4: 37.4% in the standard arm vs. 27.8% in the FEops^TM^ arm (*p* = 0.20). Complete LAAC: 44.0% in the standard arm vs. 61.1% in the FEops^TM^ arm (*p* = 0.03). Use of ≥two devices: 16.5% in the standard arm vs. 3.0% in the FEops^TM^ arm (*p* < 0.01). Repositioning a device > three times: 22.7% in the standard arm vs. 10.0% in the FEops^TM^ arm (*p* = 0.02).
Bavo et al. [63] (2020)	Retrospective validation study N = 30 Patients with pre- and post-procedural CT scans who received LAAO with either Amulet^TM^ (*n* = 15) or Watchman^TM^ devices (*n* = 15)	Comparison of device frame deformation parameters and LAA wall apposition in virtually implanted devices and acute implants	R2 ≥ 0.91 for all measures of device frame deformation when comparing the FEopsTM model and post-procedural CT scans. A comparison of predicted device leak and actual contrast leak on CT had a sensitivity of 81% and a specificity of 77%.
Ranard et al. [66] (2022)	Retrospective study N = 22 Patients who received LAAO with Watchman FLX^TM^ and had pre- and post-CT scans, as well as TEE imaging, available	Comparison of blinded and unblinded FEops^TM^ simulation results based on 3D-TEE measures of device deformation	Blinded FEops^TM^ simulation results included the final implanted device size in 16/22 patients (72.7%). r ≥ 0.90 for all device deformation measurements.

**Table 3:** Key findings of studies that have evaluated CFD after LAAO.

Publication	Study Design	Methodology	Key Findings
Mill et al. [73] (2021)	N = 6 (3 with DRT and 3 without DRT) Patients with Amplatzer Amulet^TM^ LAAO	CT scans were acquired twice—once between months 1 and 3 and once between months 3 and 6 after LAAC. Slicer 4.10.1 software was used to construct a 3D model. CFD simulations were performed using Ansys^TM^ Fluent 19 R3. Post-processing and visualization of the simulation results were performed using ParaView 5.4.1.	ECAP indices ≥ 0.5 Pa − 1 near the device surface were observed in all patients with DRT. The average velocity at the device surface: 0.15 m/s for the DRT group compared to 0.20 m/s for those without DRT. Low flow velocities < 0.2 m/s adjacent to the device surface and regions of high flow complexity with low wall shear stress are associated with DRT.
Aguado et al. [74] (2019)	N = 4 Patients with Watchman^TM^ or Amulet^TM^ LAAO (3 Watchman^TM^, 1 Amulet^TM^)	Surface meshes of the LA were reconstructed from 3D CT images or rotational angiography images. The VIDAA platform was used to explore multiple Watchman^TM^ and Amulet^TM^ configurations. The VIDAA data was then exported for CFD simulation using Ansys^TM^ Fluent 18.2.	Higher ECAP values were apparent with misplaced LAAO or undersized LAAO devices.
Vogl et al. [75] (2022)	Retrospective, N = 4 Patients with Watchman^TM^ LAAO (2 patients with DRT and 2 without DRT)	Three-dimensional models were created based on CT imaging. FEops^TM^ was used to deploy the device. CFD was performed on patient-specific models before and after device implantation.	Wall shear stress was lower for the DRT patients compared to the control patients. An increase in the atrial velocity from pre- to post-implantation was only observed in the non-DRT patients.
D’Alessandro et al. [76] (2023)	N = 5 Simulated pre-LAAO morphology, LAAO with “pacifier device”, and LAAO with “plug device”	LA models were obtained from CT imaging.	Velocities < 0.2 m/s and ECAP indices > 0.5 Pa−1 are both risk factors that predict DRT. LAAO reduces DRT risk by increasing the blood velocity and reducing ECAP indices. LAAO with a “pacifier” device had lower ECAP indices, suggesting less risk of thrombosis.
Planas et al. [77] (2022)	N = 6 Watchman FLX^TM^ and Amulet^TM^ LAAO simulations	Three-dimensional surface meshes were created from pre-procedural CT scans using Meshmixer. VIDAA simulations were performed with the pulmonary ridge covered and uncovered. CFD was performed for all simulations using Ansys^TM^ Fluent 19.2.	Low velocities were observed more frequently with uncovered pulmonary ridge configurations. The Watchman FLX^TM^ device had higher ECAP values than the Amulet^TM^.

## Data Availability

No new data were generated or analyzed in support of this research.

## Abbreviations

The following abbreviations are used in this manuscript:

CFD	Computational fluid dynamics
FSI	Fluid–structure interaction
ECAP	Endothelial cell activation potential
VIDAA	Virtual implantation and device selection in left atrial appendages
ARR	Aortic root rupture
FEA	Finite element analysis
FEops^TM^	Finite element-optimized patient-specific
DASI	Direct analytical surgical individualization
ViR	Valve in ring
THV	Transcatheter heart valve
CO	Coronary obstruction
ViV	Valve in valve
ViMAC	Valve in mitral annular calcification
LAAO	Left atrial appendage occlusion
CPI	Contact pressure index
PDL	Peri-device leak
DRT	Device-related thrombosis
LVOT	Left ventricular outflow tract
SSR	Shear strain rate
MAC	Mitral annular calcification
m-TEER	Mitral transcatheter edge-to-edge repair
PVL	Paravalvular leak
TAVI	Transcatheter aortic valve implantation
TAVR	Transcatheter aortic valve replacement
